# circPOLR1C Promotes the Development of Esophageal Cancer by Adsorbing miR-361-3p and Regulating Cancer Cell Apoptosis and Metastasis

**DOI:** 10.1155/2022/9124142

**Published:** 2022-12-23

**Authors:** Yong Fang, Jun Yin, Yaxing Shen, Hao Wang, Han Tang, Xiaosang Chen

**Affiliations:** Department of Thoracic Surgery, Zhongshan Hospital, Fudan University, Xuhui District, Shanghai 200032, China

## Abstract

**Background:**

The effect of circular RNA-RNA polymerase I and III subunit C (circPOLR1C) on esophageal cancer (EC) has not been reported. Herein, this study is designed to unveil the effect and the regulatory mechanism of circPOLR1C on EC.

**Methods:**

The expression of circPOLR1C in EC tissues and cells was detected by qRT-PCR. Circular structure, stability, and cell localization of circPOLR1C were confirmed by qRT-PCR, RNase R, actinomycin D, and fluorescence *in situ* hybridization (FISH) assay. Cell function experiments, nude mouse xenograft, lung transplant model, and HE staining were performed to evaluate the effects of CircPOLR1C on EC *in vitro* and *in vivo*. A regulatory relationship between miR-361-3p and circPOLR1C was confirmed by qRT-PCR, circRNA *in vivo* precipitation, RIP, FISH, CircInteractome database, dual-luciferase reporter assay, and immunohistochemistry. Rescue experiments were applied to assess the effects of miR-361-3p and circPOLR1C on EC cells and tissues. Apoptosis- and epithelial-mesenchymal transformation (EMT)-related gene expressions were quantified by qRT-PCR and Western blot.

**Results:**

Highly expressed circPOLR1C in EC was related to tumor differentiation and invasion. circPOLR1C, which mainly exists in the cytoplasm, is a stable circular RNA. circPOLR1C silencing inhibited circPOLR1C expression and EC cell malignant function, while circPOLR1C overexpression promoted the growth of transplanted tumors and lung metastasis. The enrichment of miR-361-3p was higher than that of other targeted miRNAs. circPOLR1C adsorbed miR-361-3p to regulate apoptosis- and EMT-related genes and partially reversed the tumor suppressive effect of miR-361-3p, which was lowly expressed in EC tissues. Silencing the target genes of miR-361-3p also inhibited the malignant development of EC cells.

**Conclusion:**

circPOLR1C adsorbs miR-361-3p and regulates apoptosis- and EMT-related gene expressions to promote the development of EC.

## 1. Introduction

Esophageal cancer (EC) occupies the 8th place in the cancer incidence worldwide and ranks 6th in the global cause of cancer death [1]. Therefore, EC has become a major public health problem, greatly threatening the life of people. According to the tumor registration data in China, the incidence of EC ranks 5th in the country's total cancer incidence [2]. Besides, the 2012 survey data showed that China has the largest number of new cases of EC in the world [3]. The histological types of EC are mainly divided into esophageal squamous cell carcinoma (ESCC) and esophageal adenocarcinoma. Since the 1970s, the incidence of ESCC in many Western countries has shown a downward trend, whereas the incidence of esophageal adenocarcinoma has rapidly increased and become one of the fastest growing malignant tumors [4, 5]. Therefore, fathoming the pathogenesis of EC, finding targets for early diagnosis and intervention, and probing into prognostic biomarkers are of great significance for the prevention and treatment of EC.

Similar to most tumors, EC is a complex disease that is involved in multiple factors, multiple genes, and multiple stages of development. The molecular mechanism of its occurrence and development has not been fully elucidated [6]. In exploring the pathogenesis of EC, in addition to oncogenes, tumor suppressor genes, cytokines, and other regulatory factors, noncoding RNA has become a new direction for researchers due to its important role in cancer [7]. Circular RNAs (circRNAs) are single-stranded RNAs with a closed-loop structure and are one of the important members of the noncoding RNA family [8]. A few studies have reported that circRNA could affect the development of EC. For instance, through *in vivo* and *in vitro* experiments, Sang et al. confirmed that circRNA ciRS-7 promotes the development of ESCC by sponging miR-876-5p to upregulate MAGE-A signals [9]. Since the high expression of circGSK3*β* has been found to be positively correlated with the development of ESCC and a poor prognosis, Hu et al. recommended that the level of circGSK3*β* in plasma should be used as a biomarker for the diagnosis and prognosis of ESCC patients [10].

RNA polymerase I and III subunit C (POLR1C) is a gene encoding shared POLR1 and POLR3 subunits [11]. Studies have shown that POLR1C (hsa_circ_0076535) plays a key role in Treacher Collins syndrome and leukocyte dystrophy [12, 13]. Joseph et al. proposed that the expression of POLR1C might be related to the prognosis of patients with triple-negative breast cancer [14]. Previous studies have reported that circPOLR1C (hsa_circ_0076535) is highly expressed in EC tissues [15], but the regulatory mechanism of circPOLR1C in EC has not been reported. On the basis of the above research studies, this study further explored the role and mechanism of circPOLR1C formed by linear POLR1C cyclization in EC, so as to supplement the expression profile and function of circRNA in EC.

## 2. Methods

### 2.1. Ethical Statement

Since this study involved clinical experiments and animal experiments, the research group obtained the approval of the Ethics Committee of Zhongshan Hospital, Fudan University (No. 201701007XHK), and the Committee of Experimental Animals of Zhongshan Hospital, Fudan University (No. 201810003XHK), respectively. After consultation with patients and family members, the patients signed the informed consent form and agreed to donate samples of EC and adjacent tissues which were limited to scientific experimental research. Animal experiments strictly followed animal ethical requirements in terms of the necessity of research, the standardization of operations, and the protection of experimental animals.

### 2.2. Tissue Sample Collection

From January 2017 to December 2018, 46 patients undergoing pathological diagnosis of primary ESCC in the Department of Gastroenterology of our hospital, who had not received radiotherapy or chemotherapy before surgery, were included in this research. EC tissues and adjacent tissues (≤3 cm from the cancer tissue, about 1 cm^3^) of the patients were collected during esophagectomy and placed in the RNA locker quickly. After the operation, the tissues were stored at −20°C for later use. Later, we also analyzed the correlation of circPOLR1C expression with gender, age, tumor size (cm), differentiation, tumor invasion (T), lymph node metastasis, TNM stage, and other clinical information of patients.

### 2.3. EC Cell Culture

In order to clarify the effect of circPOLR1C on the biological function of EC cells, we purchased human esophageal normal cell line Het-1A (ATCC® CRL-2692™, American Type Culture Collection) and various EC cell lines (KYSE-30, CL-0577, Procell, China; KYSE150, CL-0638, Procell, China; KYSE-220, laboratory preserved; TE-9, laboratory preserved; and EC9706, MZ-1077, MingZhouBio, China) and carried out differential expression research. According to the instructions of the manufacturer, cells were cultured with their specific complete cell culture media as follows: Het-1A cells were cultured in BEGM™ bronchial epithelial cell growth medium (CC-3171, Lonza/Clonetics Corporation, Switzerland) containing fetal bovine serum (FBS, 10%, SFBS, Bovogen, Australia); KYSE-30, KYSE150, KYSE-220, and TE-9 cells were maintained in RPMI-1640 medium (PM150110, Procell, China) supplemented with Ham's F-12 nutrient mixture (PM150810, Procell, China), FBS (10%), and penicillin-streptomycin solution (P/S, 1%, PB180120, Procell, China); EC9706 cells were grown in high-sugar Dulbecco's modified Eagle's medium (DMEM, C11995500BT, Gibco, USA) blended with FBS (10%) and P/S (1%). All cells were cultivated in a 5% MCO-20AIC CO_2_ incubator (SANYO, Japan) at 37°C.

### 2.4. Extraction of RNA

The extraction method of RNA referred to the existing literature reports. Briefly, TRIzol (1 mL, 15596018, Invitrogen, USA) was used to separate RNA and other components from cells. After the addition of trichloromethane (200 *μ*L, T819285, Macklin, China) and subsequent centrifugation, the mixed solution was divided into aqueous (containing RNA) and organic phases. Next, isopropanol (500 *μ*L, I811932, Macklin, China) was added to recover the RNA precipitate. Thereafter, the obtained precipitate was dissolved by an appropriate amount of diethylpyrocarbonate (DEPC) water, after which RNA concentration was detected by using a Qubit™ 4 fluorometer (Q33226, ThermoFisher, USA). Later, the RNA solution was stored in a refrigerator at −80°C. To determine the specific expression site of circPOLR1C in cells, the cytoplasm and the nucleus were separated, and RNA was extracted by using the Minute ™ cytoplasmic and nuclear separation kit (SC-003, Invent, China).

### 2.5. Ribonuclease R (RNase R) Treatment

RNase R is a 3′-5′ ribonuclease derived from the E. coli RNR superfamily and has the function of cleaving RNA [16]. RNase R (R0301, Geneseed, China) treatment was required before the analysis and identification of circPOLR1C enrichment, given that RNase R could digest almost all linear RNAs, but it was not easy to digest circular RNAs. Concretely, 10 U of RNase R was added into 2.5 *μ*g total RNA, and the resulting mixture was incubated at 37°C for 30 minutes (min).

### 2.6. RNA Reverse Transcription and Quantitative Real-Time Polymerase Chain Reaction (qRT-PCR)

Invitrogen ™ M-MLV reverse transcriptase (28025021, USA) was used for the reverse transcription of RNA into cDNA. Subsequently, the TB Green® Premix Ex Taq™ II (Tli RNaseH Plus) ROX plus reagent (RR82LR) and the SmartChip™ real-time PCR system (640022) ordered from Takara (Japan) were applied to carry out qRT-PCR in the following conditions: predenaturation at 95°C for 10 min, denaturation at 95°C for 15 seconds (s), and annealing at 60°C for 1 min, for a total of 40 cycles. The primer sequences used in this study are all supplemented in Table 1. GAPDH and U6 were regarded as internal references during this research. Relative expression levels were calculated using the 2^−ΔΔCT^ method [17].

### 2.7. Actinomycin D Transcription Suppression Experiment

Actinomycin D was employed to inhibit RNA synthesis and interfere with the cell transcription process. In a nutshell, EC cells were seeded in 24-well plates at a density of 5 × 10^4^ cells/well, and 5 test wells were set up in each group. Then, 2 *μ*g/ml actinomycin D (HY-17559, MedChemexpress, USA) was added to the cells. After addition of actinomycin D, RNA in the cells was extracted at 0 hour (h), 4 h, 8 h, 12 h, and 24 h and detected by qRT-PCR.

### 2.8. RNA Fluorescence *In Situ* Hybridization (FISH)

A biotin-labeled miR-361-3p probe (biotin-GGGGGTCCACACTAAGACT-biotin) and a digoxin-labeled circPOLR1C probe (circPOLR1C, digoxin-TCCGGAATCTACATTTATTTGAGTGGGTTTGTTGTGCCTCGTCCCGATGATCCTGCGTG-digoxin) synthesized by Beijing Abace Biology Co., Ltd. were added into EC cells. The biotin-labeled miR-361-3p probe could be detected by Cy5-streptavidin (016-170-084, Jackson, USA) with red color. Similarly, the digoxin-labeled circPOLR1C probe could be determined by using the Alexa Fluor™ 488 Tyramide SuperBoost™ kit (B40932, Invitrogen, USA) with green color. Besides, nuclei could be stained by 4,6-diamidino-2-phenylindole (DAPI). After that, the final experimental result was observed and analyzed by using a Leica DMi8 inverted fluorescence microscope (Germany) under 400 × magnification.

### 2.9. Transfection

Guangzhou Geneseed Biological Company (China) provided plasmids of overexpressed circPOLR1C, small interfering RNA for circPOLR1C (sicircPOLR1C; CCCAAACAACACGGAGACGGA), small interfering RNA for dual specificity phosphatase 2 (siDUSP2), and small interfering RNA for an X-linked inhibitor of apoptosis (siXIAP) and silenced BCL2 apoptotic regulator (siBCL2), as well as their negative controls. The miR-361-3p mimic (M; miR40004682-4-5) and the miR-361-3p inhibitor (I; miR30004682-4-5) were purchased from RIBOBIO. Next, the Lipofectamine 3000 kit (L3000008, ThermoFisher, USA) was used to transfect the plasmids into cells. 48 h after cell transfection, transfection efficiency was detected by qRT-PCR.

### 2.10. circRNA *In Vivo* Precipitation (circRIP)

The circRIP assay was conducted to purify and analyze RNA or miRNA related to circPOLR1C. Concretely, biotin-labeled DNA probes (Shanghai Biotechnology Co., Ltd.; probe sequences, TACATTTATTTGAGTGGGTTTGTTGTGCCTCGTCCCGATGATCCTGCGTGCGCGCTCT-biotin), which could specifically bind circPOLR1C, were transfected into EC cells. 24 h later, the cells were washed with PBS and fixed by 1% formaldehyde for 10 min. Next, the cells were treated by 1 mL of RIPA lysis and extraction buffer (89901, Thermo Scientific™, USA) and then placed in an ultrasonic disruption instrument (SM-150D, Shunma Tech, China) for 10 min. Thereafter, the supernatant was collected and then incubated with Dynabeads™ M-280 streptavidin (11205D, Invitrogen™, USA) at 30°C for 12 h. Ultimately, the mixed solution was further processed with Invitrogen™ proteinase K solution (AM2546, USA), followed by RNA extraction, reverse transcription, and qRT-PCR.

### 2.11. RNA Immunoprecipitation (RIP)

RIP was the use of antibodies against the target protein to precipitate the target protein and the RNA bound to it. After separation and purification, RNA in the complex could be detected and analyzed by qRT-PCR. As described in the relevant literature [18], we used the Geneseed RNA immunoprecipitation kit (P0101, China) to complete detection. circANRIL was used as a negative control in the experiment and did not bind to the Argonaute-2 (AGO2) antibody (ab32381, Abcam, UK).

### 2.12. Cell Counting Kit (CCK)-8 Assay

EC cells were digested and seeded in 96-well plates at a density of 1 × 10^4^ cells/well. Next, 10 *μ*L of the CCK-8 reagent was slowly added to the bottom of the 96-well plate containing cancer cells in order to prevent air bubbles. Then, the cells were incubated at 37°C with 5% CO_2_ and subsequently detected by using a SpectraMax® Paradigm® multifunctional microplate reader (Molecular Devices, USA) at a wavelength of 450 nm. Finally, the optical density (OD) values were recorded at 0 h, 24 h, and 48 h.

### 2.13. Cell Clone Formation Assay

EC cells transfected with sicircPOLR1C or the miR-361-3p mimic were first digested with Trypsin-EDTA solution (0.25%, 25200072, GIBCO, USA) and then made into cell suspension with a concentration of 3 × 10^4^ cells/mL. Thereafter, methanol fixation (15 min) and Giemsa (G4507-5G, Sigma, USA) staining (20 min) were carried out. Eventually, the fully dried cells were placed under a Leica DMi8 microscope (Germany) for observation of the formed cell colonies in order to detect cell proliferation.

### 2.14. Scratch Test

Predigested EC cells (3 × 10^5^ cells/well) were seeded on 12-well plates.After incubation in an MCO-20AIC CO_2_ incubator at 37°C for 24 h, a sterile pipette was applied to vertically draw parallel lines (1 cm apart) in the plate. Next, PBS was used to wash floating cells and debris off the plate. Subsequently, cell culture fluid without serum was added to the corresponding cells. After 48 h, the 12-well plates containing the cells were again observed and photographed under a microscope at a magnification of 100x. Ultimately, the migration rate was calculated to assess the effects of different intervention conditions on EC cell migration.

### 2.15. Transwell Assay

According to the manufacturer's instructions, the Transwell chamber with 8 *μ*m pore polycarbonate membrane insert and Matrigel purchased from BD Biosciences (USA) were installed and added, respectively. In a nutshell, EC cells (5 × 10^4^ cells/well) were inoculated into the upper chamber of the device covered with Matrigel, and the lower chamber was supplemented with complete medium containing FBS. 48 h later, the cells were harvested, fixed in paraformaldehyde (4%) for 30 min, and stained with crystal violet (0.1%) for 10 min. In the end, the change in cell invasion was observed by using a Leica DMi8 microscope (Germany) under 250 × magnification, followed by calculation of the cell invasion rate.

### 2.16. Flow Cytometry

Apoptosis, as one of the important basic biological functions of cells, was detected by flow cytometry. In brief, 1 × 10^5^ EC cells were inoculated into 500 *μ*L diluted 1 × Annexin V binding buffer working solution according to the instructions of the Annexin V-FITC/PI fluorescence double staining apoptotic detection kit (P-CA-201, Procell, China), followed by being mixed into a cell suspension. Subsequently, 5 *μ*L of Annexin V-FITC and 5 *μ*L of propidium iodide (PI) staining solution were added to the EC cell suspension in sequence. The mixture was then blended and incubated at room temperature for 20 min. After the reaction, the liquid was transferred to a DxFLEX flow cytometer (Backman Coulter, USA) for further analysis and detection.

### 2.17. Tumor Formation Experiment and Metastasis Experiment of Nude Mice

72 male BALB/C nude mice (6 weeks old, weight 18 ± 1 g) used in the experiment were all purchased from the Guangdong Medical Laboratory Animal Center (GDMLAC). The experimental animals were kept in the SPF-level experimental animal center, and they could eat and drink freely. 48 nude mice were randomly divided into 8 groups (6 mice/group) according to body weight after 3 days of feeding. Mice in the NC + MC group were injected with KYSE-30 or EC9706 cells transfected with the negative control and miR-361-3p mimic control; mice in the circPOLR1C + MC group were injected with KYSE-30 or EC9706 cells transfected with the circPOLR1C overexpression plasmid and the miR-361-3p mimic control; mice in the NC + M group were injected with KYSE-30 or EC9706 cells transfected with the negative control and the miR-361-3p mimic; mice in the circPOLR1C + M group were injected with KYSE-30 cells or EC9706 cells transfected with the overexpression plasmid of circPOLR1C and the miR-361-3p mimic. 1 × 10^5^ cells were injected subcutaneously into the left armpit of each nude mouse. In order to ensure the timeliness of transfected cells, we supplemented the injection once a week. Tumor volume (mm^3^, long diameter × long diameter × short diameter/2) was measured with a vernier caliper every 5 days. All animals were sacrificed on the 35th day after injection, and after that, the transplanted tumor was removed, photographed, and weighed.

BALB/C nude mice were also used in the lung metastasis experiment. 24 nude mice were randomly divided into 8 groups (3 mice/group) according to the same grouping principle as mentioned before. The lung transplantation model was constructed by an injection of KYSE-30 or EC9706 cells (1 × 10^5^ cells) via the tail vein of mice. After supplementary injection in the same way as mentioned before (once a week), all nude mice were sacrificed in the 8th week after injection and the lung tissue was collected.

### 2.18. Hematoxylin-Eosin (HE) Staining

Transplanted tumors were fixed in 4% paraformaldehyde, gradiently dehydrated, and embedded in paraffin to make paraffin sections. Dewaxed and hydrated sections were immersed in the hematoxylin reagent (B25380-20 mg, YuanYe, China) and eosin reagent (G1100-500, Solarbio, China) successively. After staining, slices were again dehydrated with gradient alcohol and observed under a Leica DMi8 fluorescence microscope at a magnification of 100 times.

### 2.19. Target Prediction and Verification

CircInteractome (https://circinteractome.nia.nih.gov/index.html) was utilized to predict miRNAs that could bind to circPOLR1C and to identify binding sites. Additionally, the starBase database (https://starbase.sysu.edu.cn/) was applied to analyze the target genes with binding sites for miR-361-3p. Then, the dual-luciferase reporter assay was conducted to detect the specific sequence binding of transcription factors and their target promoters. Wild-type (WT: circPOLR1C-WT, DUSP2-WT, XIAP-WT, and BCL2-WT) and mutant-type (MUT: circPOLR1C-MUT, DUSP2-MUT, XIAP-MUT, and BCL2-MUT) reporter plasmids were constructed using pmirGLO plasmids (CL414-01, Biomed, China). Thereafter, the abovementioned reporter plasmids were cotransfected with the miR-361-3p mimic (mimic control) or miR-361-3p inhibitor (inhibitor control) into EC cells for 24 h, respectively. Later, 150 *μ*L 1 × passive lysis buffer and 50 *μ*L Luciferase assay reagent II were added to the transfected EC cells in sequence, and after that, the mixed solution was tested twice by Promega GloMax. Specifically, firefly luciferase activity was detected in the first part. In the second part, after the former fluorescence reaction was stopped by Stop & Glo, *Renilla* luciferase activity was determined. Ultimately, the final report of plasmid activity was obtained by calculating the firefly/*Renilla* ratio.

### 2.20. Western Blot

Western blot was divided into steps of sample preparation, electrophoresis, membrane transfer, antigen-antibody binding, and color development [19]. In brief, 100 *μ*L of RIPA lysis buffer (P0013 K, Beyotime, China) was added to the preprepared EC cells or tissue homogenates to help cells fully lyse. Then, protein concentration was determined by using the Pierce ™ Rapid Gold BCA protein assay kit (A53225, Thermo Scientific™, USA) and the SpectraMax® Paradigm® microplate reader. Thereafter, 40 *μ*g of protein was separated by sodium dodecyl sulfate--polyacrylamide gel electrophoresis (SDS-PAGE) and transferred into a PVDF membrane (IPVH00010, USA) purchased from Millipore. Subsequently, the membrane was blocked with Blocker™ FL fluorescent blocking buffer (37565, Thermo Scientific™, USA) for 2 h. The process of antigen-antibody binding includes two parts: the primary antibody and the antigen in proteins were bound at 4°C overnight, and the secondary antibody was recognized by the primary antibody in proteins at room temperature for 1.5 h. Next, ECL luminous fluid (WBKlS0100, Millipore, USA) and the Gel Doc™ XR + system (BIO-RAD, USA) were exploited for the final color development process. Herein, all the antibodies used in Western blot were uniformly purchased from Abcam, UK. Primary antibodies included those against cleaved caspase-3 (ab2302, 17KD, 1 : 1000), Bcl-2 (ab59348, 26KD, 1 : 500), Bax (ab32503, 21KD, 1 : 1000), E-cadherin (ab40772, 97 KD, 1 : 10000), N-cadherin (ab18203, 130KD, 1 : 1000), vimentin (ab92547, 54 KD, 1 : 2000), dual specificity phosphatase 2 (DUSP2, ab224407, 24KD, 1 : 2000), an X-linked inhibitor of apoptotic protein (XIAP, ab2541, 54KD, 1 : 1000), and GAPDH (internal reference, ab8245, 36KD, 1 : 5000). Secondary antibodies included a goat antirabbit antibody (ab205718, 1 : 5000) and a goat antimouse antibody (ab150117, 1 : 5000).

### 2.21. Immunohistochemistry

Paraffin sections of transplanted tumor tissues made before were permeated in the Triton X-100 reagent (0.01%, 10 min, KGF011, Keygen, China). Next, the H_2_O_2_ reagent (3%, 10 min) and goat serum (10%, 1 h, C0265, Beyotime, China) were employed to treat slices, respectively. Then, anti-Ki67 antibody [SP6] (ab16667, Abcam, UK) diluted 250-fold was added dropwise to incubate the tissue slices at 4°C overnight. On the next day, the SABC-POD immunohistochemistry kit (I001-2-1, Nanjing Jiancheng, China) was applied to stain the sections. Later, the stained sections were dehydrated, transparentized, and mounted. Finally, the color development of the slices was observed using the microscope at a magnification of 400 times to evaluate the positive expression of Ki-67.

### 2.22. Data Analysis

Data analysis was conducted using SPSS 22.0 software (USA). Student's two-tailed*t*-test was applied to compare differences between two groups, and one-way analysis of variance (ANOVA) was utilized to contrast differences among multiple groups. The difference was determined to be statistically significant at *p* < 0.05.

## 3. Results

### 3.1. Characteristics of circPOLR1C in EC

**Figure 1:**
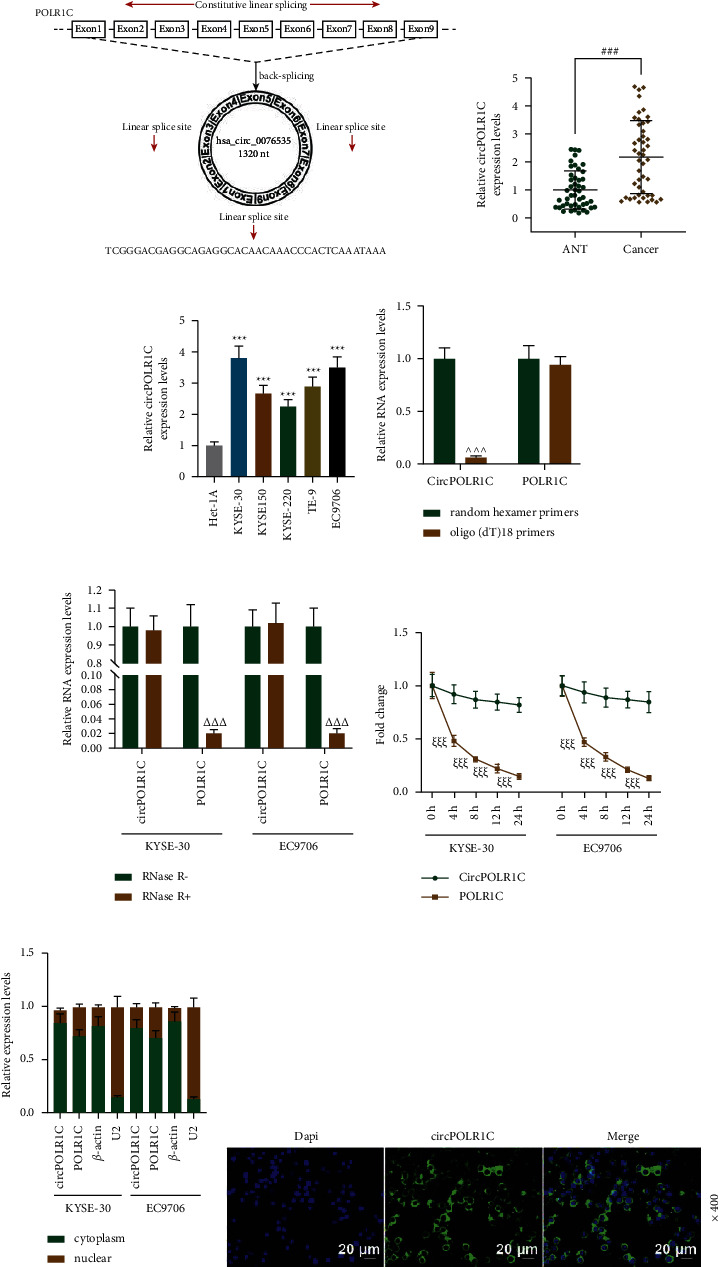
Characteristics of circPOLR1C in EC. (a) The structure of circPOLR1C. (b) The expression of circPOLR1C in EC tissues and adjacent tissues was detected by quantitative real-time polymerase chain reaction (qRT-PCR) (*n* = 46). (c) The expression of circPOLR1C in EC cells and normal human esophageal cells was quantified by qRT-PCR. (d) Random hexamer primers and oligo(dT) 18 primers were used to analyze the expressions of circPOLR1C and linear POLR1C by qRT-PCR. (e) The expression stability of circPOLR1C was investigated by the RNase R experiment. (f) 0 h, 4 h, 8 h, 12 h, and 24 h after the addition of actinomycin D, the half-lives of circPOLR1C and linear POLR1C were detected by qRT-PCR. (g) The expression of circPOLR1C in EC cells was measured by cytoplasmic nuclei isolation and qRT-PCR. *β*-Actin and U2 acted as internal references. (h) The expression of circPOLR1C in EC cells was tested by RNA fluorescence *in situ* hybridization (FISH) under 400 × magnification. All experiments were independently performed in triplicate. ^###^*p* < 0.001 vs. adjacent tissue (ANT); ^*∗∗∗*^*p* < 0.001 vs. Het-1A; ^ ^ ^*p* < 0.001 vs. random hexamer primers; ^ΔΔΔ^*p* < 0.001 vs. RNase R-; ^*ξξξ*^*p* < 0.001 vs. circPOLR1C.

The structure of circPOLR1C is presented in Figure 1(a). We first detected the differential expression of circPOLR1C in EC tissues and cells. The results showed that the expression of circPOLR1C in EC tissues was significantly higher than that in adjacent normal tissues (Figure 1(b), *p* < 0.001), and circPOLR1C was highly expressed in EC cells compared to that in Het-1A cells (Figure 1(c), *p* < 0.001). In the study of the correlation between circPOLR1C and the clinical characteristics of patients, we found that circPOLR1C expression was only correlated with differentiation and tumor invasion (Table 2, *p* < 0.01).

We further verified the circular structure of circPOLR1C and analyzed the resistance, stability, and localization of RNase R in circPOLR1C. First, we used random hexamer primers and oligo(dT) 18 primers through qRT-PCR to analyze the expressions of circPOLR1C and linear POLR1C. Subsequently, we discovered that oligo(dT) 18 primers led to obvious downregulation of circPOLR1C but barely affected linear POLR1C expression, compared with random hexamer primers (Figure 1(d), *p* < 0.001). This result proved that circPOLR1C did not have a poly (A) tail (a characteristic of linear molecules). Experiments on the stability of circPOLR1C showed that after RNase R treatment, the level of POLR1C markedly decreased, while the level of circPOLR1C remained unchanged (Figure 1(e), *p* < 0.001). After treatment with actinomycin D, the half-life of circPOLR1C was remarkably longer than that of linear POLR1C (Figure 1(f), *p* < 0.001). This part of the results fully demonstrated that circPOLR1C was a stable and nondegradable RNA molecule. Finally, the results of the qRT-PCR analysis and FISH experiments on circPOLR1C after nucleoplasm separation are shown in Figures 1(g) and 1(h). It turned out that circPOLR1C mainly existed in the cytoplasm of EC cells.

### 3.2. Effects of sicircPOLR1C on circPOLR1C Expression, Viability, Proliferation, Migration, Invasion, and Apoptosis of EC Cells

**Figure 2:**
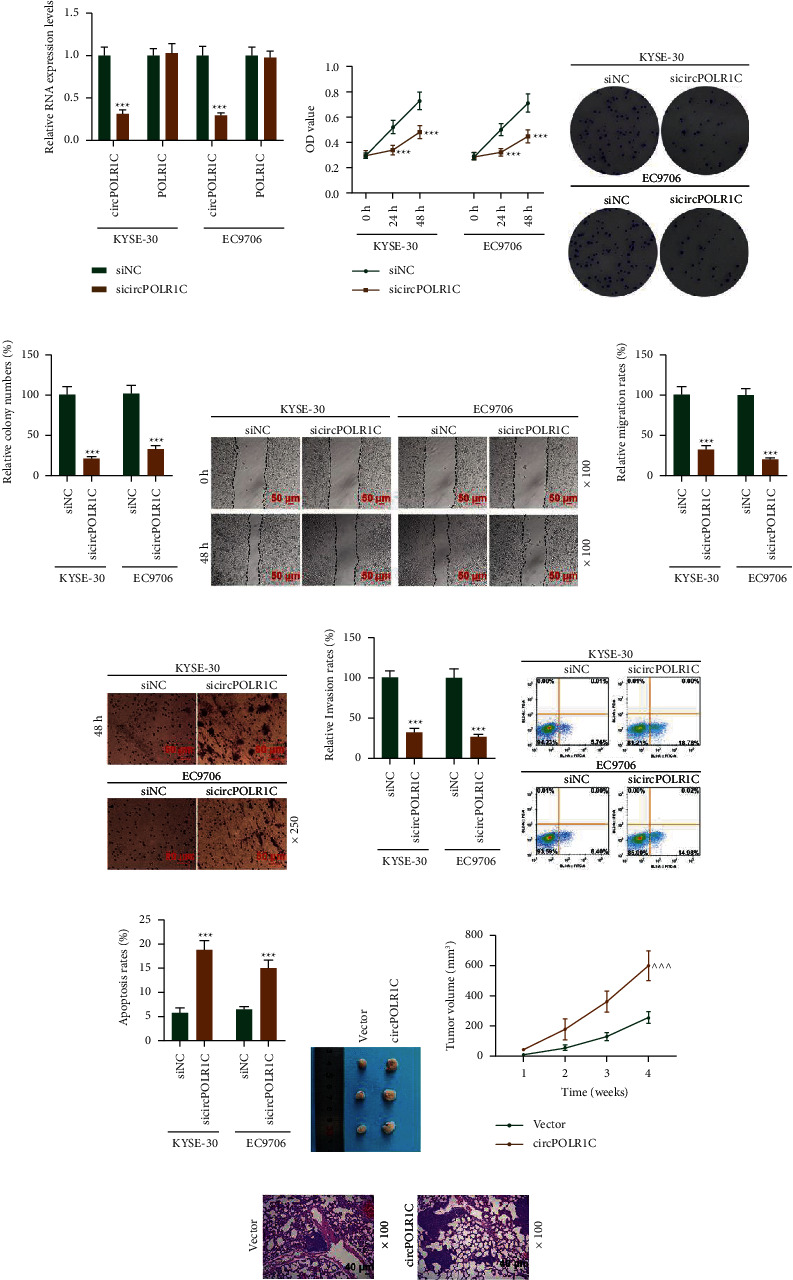
Effects of sicircPOLR1C on the viability, proliferation, migration, invasion, and apoptosis of EC cells *in vitro* and EC xenografts and lung metastasis *in vivo*. (a) The transfection efficiency of sicircPOLR1C was detected by qRT-PCR. GAPDH was applied as the internal reference. (b) The effect of sicircPOLR1C on the viability of EC cells was evaluated by the cell counting kit (CCK)-8 assay. (c, d) The effect of sicircPOLR1C on the proliferation of EC cells was assessed by the cell clone formation assay. (e, f) The effect of sicircPOLR1C on the migration of EC cells was detected by the scratch assay under 100 × magnification. (g, h) The effect of sicircPOLR1C on the invasion of EC cells was determined by the transwell assay under 250 × magnification. (i, j) The effect of sicircPOLR1C on the apoptosis of EC cells was evaluated by flow cytometry. (k, l) The effect of overexpressed circPOLR1C on the growth of EC xenografts, *n* = 6/group. (m) The effect of overexpressed circPOLR1C on the metastasis of EC was demonstrated by hematoxylin-eosin (HE) staining under 100 × magnification. All experiments were independently conducted in triplicate. ^*∗∗∗*^*p* < 0.001 vs. siNC; ^ ^ ^*p* < 0.001 vs. vector.

After sicircPOLR1C was transfected with KYSE-30 and EC9706 cells, sicircPOLR1C explicitly reduced the expression of circPOLR1C, while it had no effect on linear POLR1C (Figure 2(a), *p* < 0.001). In addition, the effects of sicircPOLR1C on the viability, proliferation, migration, invasion, and apoptosis of EC cells were analyzed by the CCK-8 assay, clone formation assay, scratch test, transwell assay, and flow cytometry method. When compared with those in the siNC group, the OD value (Figure 2(b)), colony number (Figures 2(c) and 2(d)), migration rates (Figures 2(e) and 2(f)), and invasion rates (Figure 2(g) and 2(h)) of KYSE-30 and EC9706 cells decreased in the sicircPOLR1C group (*p* < 0.001), while the apoptotic rates of KYSE-30 and EC9706 cells increased in the sicircPOLR1C group (Figures 2(i) and 2(j), *p* < 0.001). These findings indicated that sicircPOLR1C apparently impeded the viability, proliferation, migration, and invasion of EC cells while promoting the apoptosis of KYSE-30 and EC9706 cells.

### 3.3. Effect of Overexpressed circPOLR1C on EC-Transplanted Tumors and Lung Tissues

In order to further figure out the impact of circPOLR1C on EC, we constructed a nude mouse model of EC transplantation and lung metastasis. By recording the tumor volume, we found that overexpressed circPOLR1C increased the tumor volume and promoted the growth of transplanted tumors (Figures 2(k) and 2(l), *p* < 0.001). Not only that, we observed through HE staining of lung tissues that the vacuole structure notably increased in the circPOLR1C group when compared with that in the vector group, signifying that the lung tissues were obviously damaged after transfection of overexpressed circPOLR1C plasmids (Figure 2(m)). Moreover, the *in vivo* experimental results showed that overexpressed circPOLR1C promoted the growth of EC xenografts and potentiated lung tissue destruction.

### 3.4. circPOLR1C Could Act as a Sponge for miR-361-3p and Negatively Regulated miR-361-3p That Was Lowly Expressed in EC Tissues

**Figure 3:**
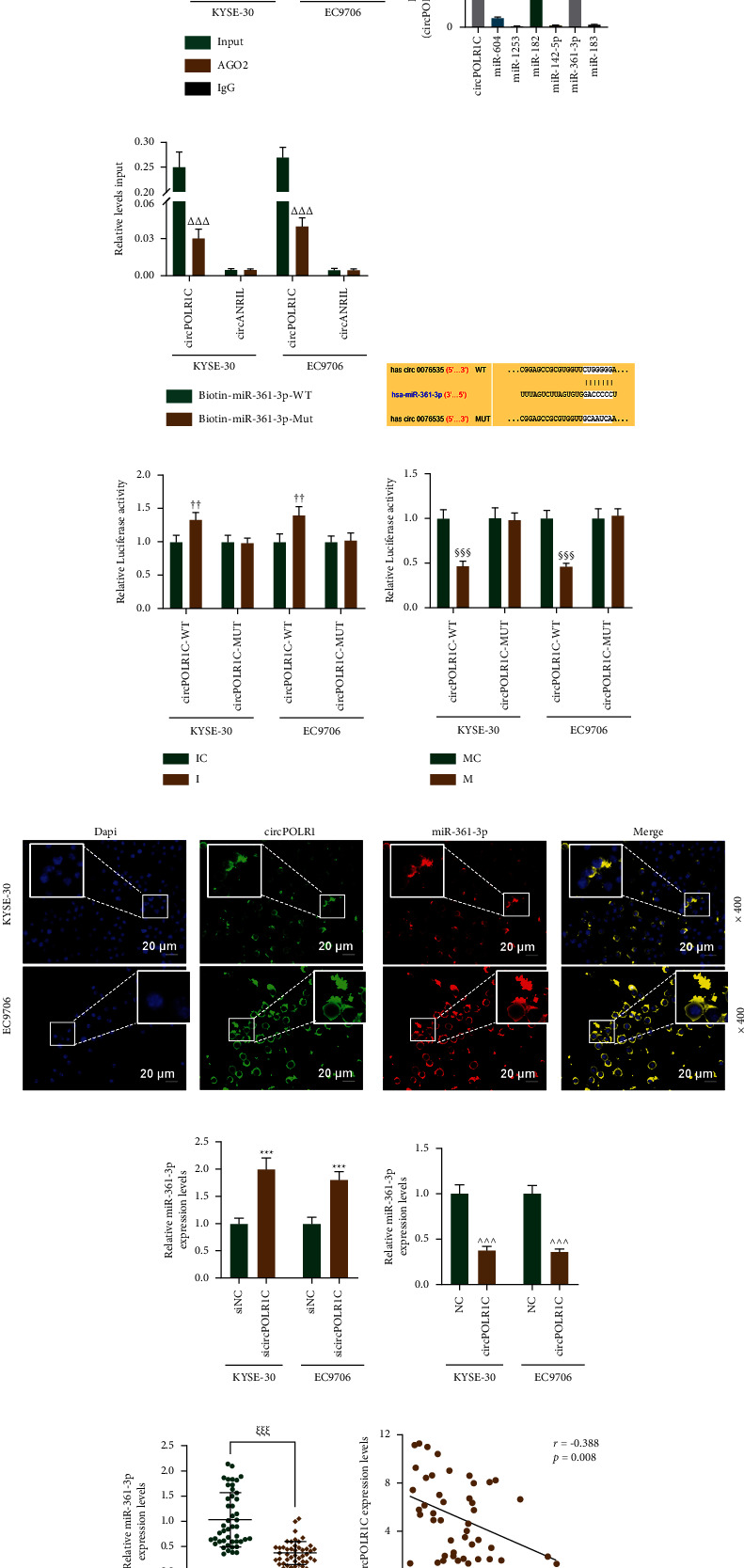
circPOLR1C could act as a sponge for miR-361-3p and negatively regulated miR-361-3p, which was poorly expressed in EC tissues. (a) Whether circPOLR1C could bind to miRNA was tested by RNA immunoprecipitation (RIP). circANRIL was employed as a negative control. (b) The enrichment of circPOLR1C and its targeted miRNAs (top 6) was detected by the circRIP experiment. (c) The enrichment of circPOLR1C and miR-361-3p was analyzed by the biotin-labeled microRNA capture experiment. (d) The targeted binding sites of circPOLR1C and miR-361-3p in CircInteractome. (e, f) The dual-luciferase reporter assay was used to verify the targeting relationship between circPOLR1C and miR-361-3p. (g) Colocalization of circPOLR1C and miR-361-3p in cells was detected by the FISH experiment under 400 × magnification. (h, i) The effects of sicircPOLR1C and overexpressed circPOLR1C on miR-361-3p expression. U6 was utilized as the internal reference. (j) The expression of miR-361-3p in EC tissues and ANT was analyzed by qRT-PCR (*n* = 46). (k) Correlation analysis of circPOLR1C and miR-361-3p in EC. All experiments were independently performed in triplicate. ^###^*p* < 0.001 vs. AGO2; ^ΔΔΔ^*p* < 0.001 vs. biotin-miR-361-3p-WT; ^††^*p* < 0.01 vs. IC; ^*§§§*^*p* < 0.001 vs. mimic control (MC); ^*∗∗∗*^*p* < 0.001 vs. siNC; ^ ^ ^*p* < 0.001 vs. NC; ^*ξξξ*^*p* < 0.001 vs. ANT.

In view of the fact that the reported circRNA could serve as a miRNA sponge, we proved by RIP that circPOLR1C could be prominently enriched by the anti-AGO2 antibody (Figure 3(a), *p* < 0.001) and could serve as a binding platform for AGO2 and miRNAs. Among the miRNAs predicted by CircInteractome that circPOLR1C might target, we selected the first 6 miRNAs and analyzed their enrichment by circRIP. As shown in Figure 3(b), we observed that probes specifically targeting circPOLR1C could enrich circPOLR1C, miR-361-3p, and miR-182, and miR-361-3p was enriched more obviously than miR-182.

To further verify the direct binding of miR-361-3p and circPOLR1C, we compared the differences in the enrichment of circPOLR1C and circANRIL (negative control) in KYSE-30 and EC9706 cells using biotin-labeled miR-361-3p-WT and biotin-labeled miR-361-3p-MUT. It turned out that biotin-labeled miR-361-3p-WT could enrich circPOLR1C rather than circANRIL (Figure 3(c), *p* < 0.001). In Figure 3(d), the binding sites of circPOLR1C and miR-361-3p are exhibited. Additionally, through the dual-luciferase reporter assay, we found that the luciferase activity of KYSE-30 and EC9706 cells increased in the circPOLR1C-WT + I group relative to that in the circPOLR1C-WT + IC group, while there was no change in luciferase activity of KYSE-30 and EC9706 cells between the circPOLR1C-MUT + IC group and circPOLR1C-MUT + I group (Figure 3(e), *p* < 0.01). Besides, the luciferase activity of KYSE-30 and EC9706 cells decreased in the circPOLR1C-WT + M group compared with that in the circPOLR1C-WT + MC group (Figure 3(f), *p* < 0.01). These results proved that miR-361-3p could bind with circPOLR1C. Furthermore, the results of the FISH experiment directly demonstrated the colocalization of circPOLR1C and miR-361-3p (Figure 3(g)).

In the results of the experiments on the mutual regulation of miR-361-3p and circPOLR1C, sicircPOLR1C was found to upregulate the expression of miR-361-3p in EC cells (Figure 3(h), *p* < 0.001), whereas overexpressed circPOLR1C produced opposite effects (Figure 3(i), *p* < 0.001). As shown in Figures 3(j) and 3(k), the expression of miR-361-3p was explicitly lower in EC tissues than that in adjacent tissues (Figure 3(j), *p* < 0.001) and miR-361-3p expression was negatively correlated with the expression of circPOLR1C in EC tissues (Figure 3(k), *p* < 0.01, *r* = −0.388).

### 3.5. Effects of the miR-361-3p Mimic and circPOLR1C Overexpression on miR-361-3p Expression, Viability, Proliferation, Migration, Invasion, and Apoptosis of EC Cells

**Figure 4:**
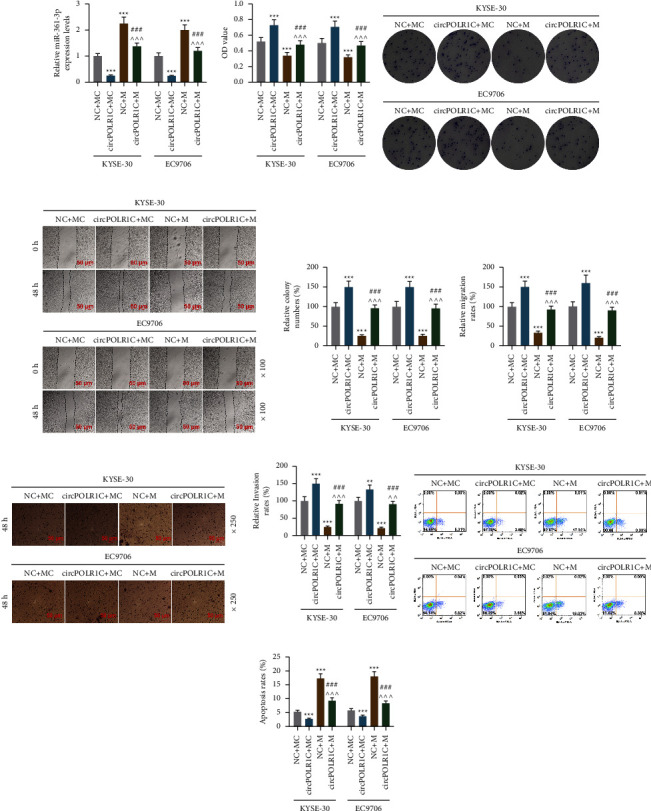
The effects of the miR-361-3p mimic and circPOLR1C on the miR-361-3p expression, viability, proliferation, migration, invasion, and apoptosis of EC cells. (a) The effects of the miR-361-3p mimic and circPOLR1C on miR-361-3p expression were evaluated by qRT-PCR. U6 was exploited as the internal reference. (b) The effects of the miR-361-3p mimic and circPOLR1C on the viability of EC cells were detected by the CCK-8 assay. (c and e) The effects of the miR-361-3p mimic and circPOLR1C on the proliferation of EC cells were evaluated by the cell clone formation assay. (d and f) The effects of the miR-361-3p mimic and circPOLR1C on the migration of EC cells were determined by the scratch assay under 100 × magnification. (g–h) The effects of the miR-361-3p mimic and circPOLR1C on the invasion of EC cells were evaluated by the transwell assay under 250 × magnification. (i, j) The effect of the miR-361-3p mimic and overexpressed circPOLR1C on the apoptosis of EC cells was examined by flow cytometry. All experiments were independently conducted in triplicate. ^*∗∗∗*^*p* < 0.001 vs. NC + mimic control (MC); ^ ^ ^*p* < 0.001 vs. NC + miR-361-3p mimic (M); ^###^*p* < 0.001 vs. circPOLR1C + MC.

After the miR-361-3p mimic and circPOLR1C overexpression plasmid were transfected with KYSE-30 and EC9706 cells, we examined their regulation and interactions on the viability, proliferation, migration, invasion, and apoptosis of carcinoma cells. The miR-361-3p mimic markedly upregulated the expression of miR-361-3p in EC cells, but this trend was partially reversed by circPOLR1C overexpression (Figure 4(a), *p* < 0.001). In terms of the basic biological functions of EC cells, we discovered that the miR-361-3p mimic suppressed vitality, proliferation, migration, and invasion (Figures 4(b)–4(h), *p* < 0.001) and enhanced the apoptosis of EC cells (Figures 4(i) and 4(j), *p* < 0.001), whereas circPOLR1C overexpression produced opposite regulatory effects (Figures 4(b)–4(j), *p* < 0.01). More importantly, circPOLR1C overexpression partially offset the regulatory effects of the miR-361-3p mimic on the above basic biological functions of EC cells (Figures 4(b)–4(j), *p* < 0.001).

### 3.6. Regulation of the miR-361-3p Mimic and circPOLR1C Overexpression on Apoptosis-Related Genes, Epithelial-Mesenchymal Transition (EMT)-Related Genes, and Oncogenes

In the mechanism study of miR-361-3p and circPOLR1C, we found that the miR-361-3p mimic upregulated the protein expressions of cleaved caspase-3, Bax, and E-cadherin as well as mRNA levels of Bax and E-cadherin but downregulated both protein expressions and mRNA levels of Bcl-2, N-cadherin, and vimentin (Supplementary Figures 1(a)–1(f), *p* < 0.001). Additionally. overexpression of CircPOLR1C did oppositelys, and partially reversed the effects of miR-361-3p mimic on the expressions of Bcl-2, Bax, cleaved Caspase-3, E-Cadherin, N-Cadherin and Vimentin (Supplementary Figures 1(a)–1(f), *p* < 0.01).

Through qRT-PCR, we further screened different oncogenes and apoptotic signaling pathways that miR-361-3p and circPOLR1C may be involved in, such as SH2B1, splicing factor 3B subunit 3 (SF3B3), DUSP2, cysteine-rich angiogenic inducer 61 (CYR61), XIAP, forkhead box O3 (FOXO3), and Bcl-2. The results showed that miR-361-3p markedly hampered the mRNA expressions of DUSP2, XIAP, and Bcl-2, while circPOLR1C overexpression produced opposite regulatory effects (Supplementary Figures 1(g) and 1(h), *p* < 0.001). Besides, circPOLR1C overexpression partially reversed the inhibitory impacts of the miR-361-3p mimic on the DUSP2, XIAP, and Bcl-2 expression levels (Supplementary Figures 1(g) and 1(h), *p* < 0.001). Subsequently, we further detected the abovementioned differentially expressed genes by Western blot and found that circPOLR1C overexpression promoted the protein expressions of DUSP2, XIAP, and Bcl-2, while the miR-361-3p mimic suppressed the protein expressions of DUSP2, XIAP, and Bcl-2 (Supplementary Figures 1(i) and 1(j), *p* < 0.001). Moreover, circPOLR1C overexpression partially negated the inhibitory effect of the miR-361-3p mimic on DUSP2, XIAP, and Bcl-2 expression levels (Supplementary Figures 1(i) and 1(j), *p* < 0.001). These experimental results showed that circPOLR1C overexpression could partially neutralize the regulatory effect of the miR-361-3p mimic on apoptosis- and EMT-related genes.

### 3.7. miR-361-3p Could Target DUSP2, XIAP, and BCL2

**Figure 5:**
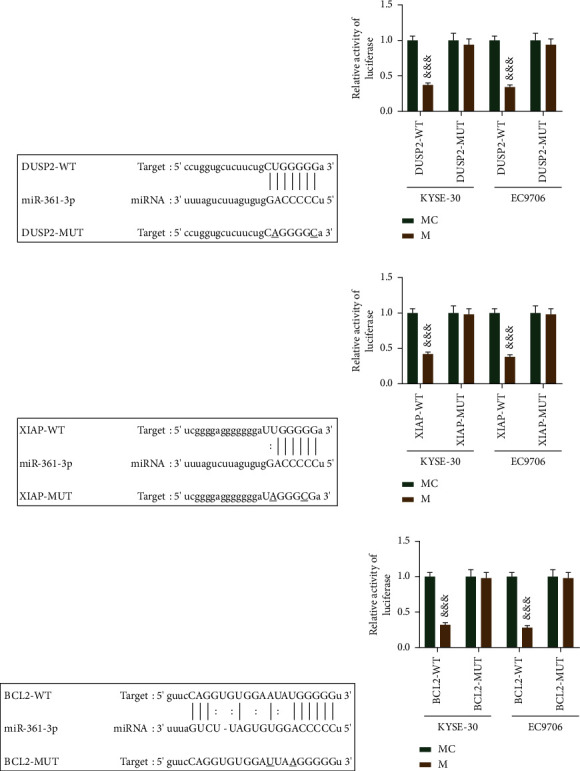
miR-361-3p could target DUSP2, XIAP, and BCL2. (a, b) Binding of DUSP2 and miR-361-3p was verified by the dual-luciferase reporter assay. (c, d) Binding of XIAP and miR-361-3p was verified by the dual-luciferase reporter assay. (e, f) Binding of BCL2 and miR-361-3p was verified by the dual-luciferase reporter assay. All experiments were independently carried out in triplicate. ^&&&^*p* < 0.001 vs. mimic control (MC).

Based on the previous research results, we further verified the binding of miR-361-3p to DUSP2, XIAP, and BCL2 by the dual-luciferase reporter assay. In cells cotransfected with the wild-type reporter genes and miR-361-3p mimic, the luciferase activity of EC cells was remarkably lower than that of control cells (Figures 5(a)–5(f), *p* < 0.001). These results showed that DUSP2, XIAP, and BCL2 could bind to miR-361-3p.

### 3.8. Effects of siDUSP2, siXIAP, and siBCL2 on the Basic Functions of EC Cells

**Figure 6:**
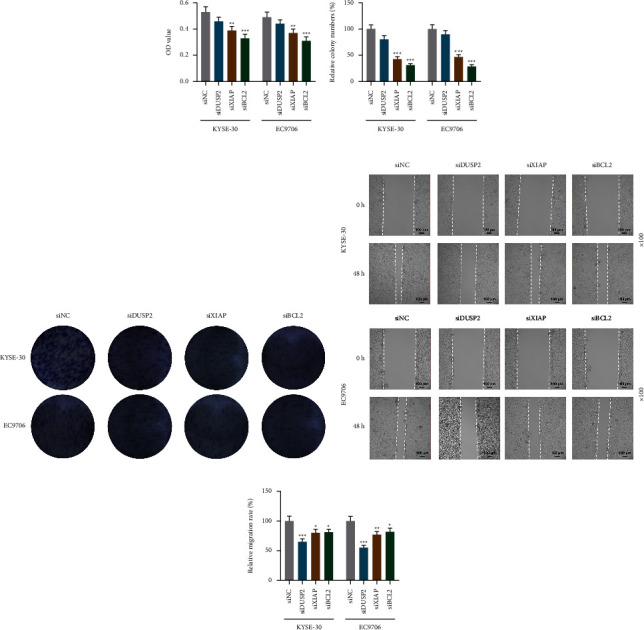
Effects of siDUSP2, siXIAP, and siBCL2 on the viability, proliferation, and migration of EC cells. (a) The effects of siDUSP2, siXIAP, and siBCL2 on the viability of EC cells were verified by the CCK-8 assay. (b, c) The effects of siDUSP2, siXIAP, and siBCL2 on EC cell proliferation were evaluated by colony formation assays. (d, e) The effects of siDUSP2, siXIAP, and siBCL2 on the migration of EC cells were tested by the scratch assay under 100 × magnification. All experiments were independently performed in triplicate. ^*∗∗*^*p* < 0.01; ^*∗∗∗*^*p* < 0.001 vs. siNC.

We further verified the effects of target genes on the basic functions of EC cells by the gene silencing pathway. As a result, we found differences in the suppressive impacts of siDUSP2, siXIAP, and siBCL2 on basic biological functions of EC cells. For example, siXIAP and siBCL2 obviously impeded the viability and proliferation of EC cells (Figures 6(a)–6(c), *p* < 0.01), but the inhibitory effect of siDUSP2 was not significant (Figures 6(a)–6(c)). For the migration and invasion of EC cells, although siDUSP2, siXIAP, and siBCL2 reduced the migration and invasion of EC cells (Figures 6(d) and 6(e) and Supplementary Figures 2(a) and 2(b), *p* < 0.05), the suppressive effect of siDUSP2 was more obvious. Moreover, silencing of the three genes increased the proportion of apoptotic EC cells, of which siXIAP generated a more significant impact (Supplementary Figures 2(c) and 2(d), *p* < 0.001).

### 3.9. Effects of the miR-361-3p Mimic and circPOLR1C on the Growth of Tumor Xenograft, Apoptosis-Related Genes, circPOLR1C, miR-361-3p, and Ki-67 Expressions *In Vivo*

**Figure 7:**
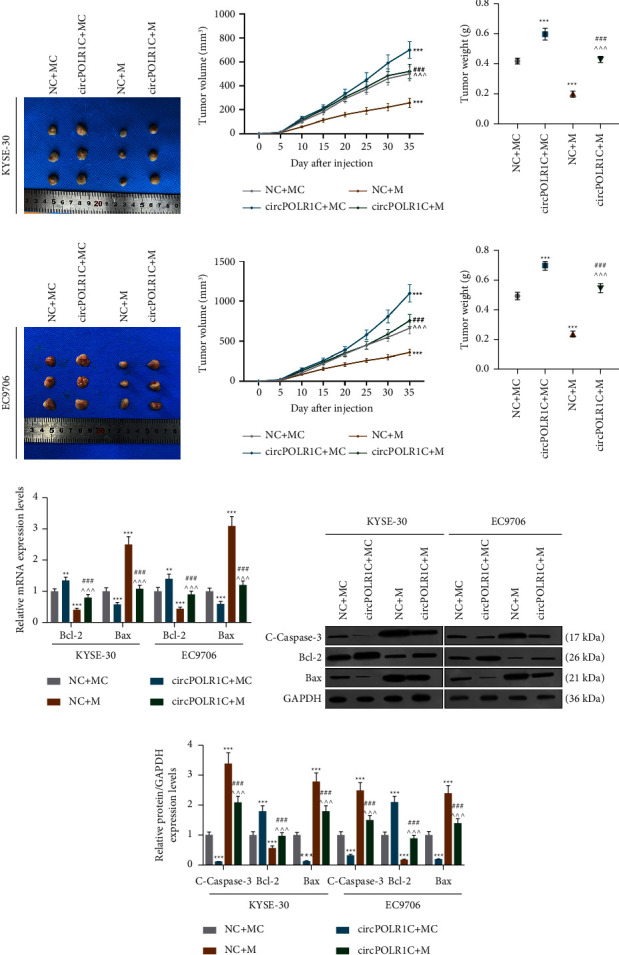
Effects of the miR-361-3p mimic and overexpressed circPOLR1C on the growth and apoptosis-related gene expressions in transplanted tumors *in vivo*. (a)–(f) The effects of the miR-361-3p mimic and circPOLR1C on the volume and weight of the transplanted tumors. (g) The effects of the miR-361-3p mimic and circPOLR1C on the mRNA levels of apoptosis-related genes were assessed by qRT-PCR. GAPDH was applied as the internal reference. (h)–(i) The effects of the miR-361-3p mimic and circPOLR1C on the protein levels of apoptosis-related genes were evaluated by Western blot. GAPDH was employed as the internal reference, *n* = 6/group. All experiments were independently carried out in triplicate. ^*∗∗*^*p* < 0.01; ^*∗∗∗*^*p* < 0.001 vs. NC + MC; ^ ^ ^*p* < 0.001 vs. NC + M; ^###^*p* < 0.001 vs. circPOLR1C + MC.

The *in vivo* experiments were designed to verify the regulatory effects of the miR-361-3p mimic and overexpressed circPOLR1C on EC. As depicted in Figure 7(a)–7(f), the miR-361-3p mimic markedly inhibited the increase in the weight and volume of transplanted tumors (*p* < 0.001), although this effect was partially reversed by overexpressed circPOLR1C (Figures 7(a)–7(f), *p* < 0.001). Similar to *in vitro* cell experiments, the miR-361-3p mimic upregulated the protein expressions of cleaved caspase-3 and Bax as well as the mRNA level of Bax but downregulated both the protein expression and mRNA level of Bcl-2 (Figures 7(g) and 7(i), *p* < 0.001). Meanwhile, overexpressed circPOLR1C partially nullified the regulatory effect of the miR-361-3p mimic on EC cells (Figures 7(g) and 7(i), *p* < 0.001).

**Figure 8:**
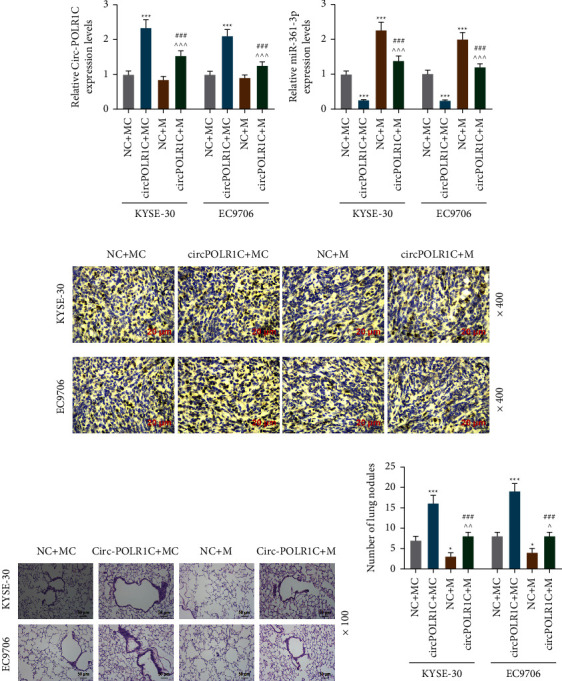
Effects of miR-361-3p and circPOLR1C on the expressions of circPOLR1C, miR-361-3p, and Ki-67*in vivo* and on the number of lung nodules in mice with lung transplantation. (a) The effects of the miR-361-3p mimic and circPOLR1C on the expression of circPOLR1C were evaluated by qRT-PCR. GAPDH was exploited as the internal reference. (b) The effects of the miR-361-3p mimic and circPOLR1C on the expression of miR-361-3p were assessed by qRT-PCR. U6 was utilized as the internal reference. (c) The effects of the miR-361-3p mimic and circPOLR1C on the expression of Ki-67 were analyzed by immunohistochemical staining under 400 × magnification. (d, e) The effects of the miR-361-3p mimic and overexpression of circPOLR1C on the number of lung nodules were evaluated by HE staining under 100 × magnification, *n* = 6/group. All experiments were independently conducted in triplicate. ^*∗*^*p* < 0.05; ^*∗∗∗*^*p* < 0.001 vs. NC + MC;  ^*p* < 0.05; ^ ^ ^*p* < 0.001 vs. NC + M; ^###^*p* < 0.001 vs. circPOLR1C + MC.

Herein, the *in vivo* findings on expression regulation of circPOLR1C and miR-361-3p in transplanted tumor tissues were also consistent with the results of *in vitro* experiments. As shown in Figures 8(a) and 8(b), the miR-361-3p mimic had no effect on the expression of circPOLR1C, whereas overexpressed circPOLR1C suppressed the mRNA expression of miR-361-3p (*p* < 0.001). Besides, the expression of Ki-67 in tissues was analyzed by immunohistochemistry. The results are shown in Figure 8(c). Compared with the NC + MC group, the brown positive expression area in the NC + M group was prominently reduced. After circPOLR1C cotransfection, the positive expression area in the circPOLR1C + M group was explicitly increased compared with that in the NC + M group.

We extracted lung tissues from lung transplantation model mice. The results of HE staining and lung nodule statistics showed that circPOLR1C overexpression increased the number of lung tissue pathological damage and lung nodules, while the miR-361-3p mimic had the opposite effect (Figures 8(d) and 8(e), *p* < 0.001). More importantly, circPOLR1C counteracted the protective effects of the miR-361-3p mimic.

## 4. Discussion

EC is a malignant tumor of esophageal origin. Although in the past few decades, great improvement has been made in the treatment of EC after combination of surgical resection, neoadjuvant chemotherapy, radiotherapy, and biological therapy. However, the lack of early diagnosis and treatment still makes the overall survival rate of EC patients low [20]. The discovery of noncoding RNAs and known biological functions provide a new direction for the prevention and treatment of EC. By dint of its special covalent closed-loop structure, circRNA protects itself from exonuclease-mediated degradation, so the expression of circRNA has high stability [8]. In this study, we further detected the expression of circPOLR1C in EC tissues and cells, probed into whether circPOLR1C was circular, and verified its stability and cell localization. As a result, we proved that circPOLR1C was abundantly expressed in EC cells and mainly located in the cytoplasm of circRNA molecules with high stability. In addition, we demonstrated that circPOLR1C, which was highly expressed in EC, had a correlation with tumor differentiation and invasion of EC cells. Furthermore, knocking down circPOLR1C expression suppressed the proliferation and metastasis yet promoted the apoptosis of EC cells, whereas upregulated circPOLR1C promoted the growth of EC xenografts and metastasis. Notably, these experimental results have not been reported in previous studies.

At present, there are few studies on circRNA intervention in EC. circRNAs, such as circRAD23B [21], ciRS-7 [22], and circRNA_100367 [23], regulate the occurrence and development of EC, whose mechanisms are reported to be mostly based on circRNA acting as an miRNA sponge. With this remarkable feature of circRNA, this study further explored whether circPOLR1C could bind miRNA. The results showed that circPOLR1C could adsorb miR-361-3p to form a competitive sponge of miR-361-3p, partially reversing the regulatory effect of miR-361-3p on EC.

Abnormal expression of miR-361-3p has been reported to be related to the proliferation and apoptosis of various tumor cells. For instance, the low expression of miR-361-3p attenuates the proliferation and metastasis of non-small-cell lung cancer cells by inhibiting the SH2B1 gene [3]. Besides, miR-361-3p, which is also lowly expressed in retinoblastoma, has been shown to exert a tumor suppressive effect by targeting SHH signaling [24]. Similar to the above research results, the expression of miR-361-3p in EC tissues was found to be decreased in this study, while overexpression of miR-361-3p can suppress the proliferation and metastasis yet promote the apoptosis of EC cells. This result suggests that miR-361-3p acts as a tumor suppressor gene in EC. In addition to cancer suppression, miR-361-3p has also been demonstrated to have a cancer-promoting effect. As previously described, miR-361-3p enhances the EMT process of pancreatic ductal adenocarcinoma cells by regulating the DUSP2/ERK axis [25]. Also, it has been reported that the cancer-promoting effect of miR-361-3p with abnormally elevated expression in breast cancer tissue is achieved by inhibiting the E2F1/P73 pathway [26]. These findings show that miR-361-3p can produce different stimulation effects in different types of cancer. Apart from the regulatory role of miR-361-3p itself, miR-361-3p is the target gene of circRNA, which is also regulated by circRNA and interferes with tumor progression. Chen et al. [27] found that circRNA 100146 binds with miR-361-3p in non-small-cell lung cancer to regulate downstream mRNA. Wang et al. [28] confirmed that as a sponge of miR-361-3p, circ-MYBL2 promotes the growth and metastasis of cervical cancer cells. hsa_circ_0011385, which also acts as a sponge and negatively regulates miR-361-3p, has a significant promoting effect on thyroid cancer [29]. These pieces of research evidence strongly support our research results that miR-361-3p, as the target gene of circPOLR1C, is adsorbed by circPOLR1C and negatively regulated. At the same time, this study demonstrated that circPOLR1C regulates EC cell apoptosis and EMT-related genes by adsorbing miR-361-3p.

Cleaved caspase-3, Bax, Bcl-2, DUSP2, and XIAP are the key genes that regulate the process of cell apoptosis. A prior study has shown that the BIR region in the XIAP structure can inhibit caspase activity and is the key to the antiapoptotic effect of XIAP [30]. Besides, overexpression of DUSP2 promotes the apoptosis of ectopic stromal cells [31]. Also, the antiapoptotic gene Bcl-2 can directly adjust the permeability of the mitochondrial membrane to prevent the release of cytochrome C into the cytoplasm, thereby inhibiting the activation of caspase-3 and preventing apoptosis [32]. On the contrary, Bax from the same family as Bcl-2 has a significant proapoptotic effect [32]. Notably, in this study, we discovered that overexpression of circPOLR1C adsorbed miR-361-3p and partially offset the effects of miR-361-3p on downregulating the levels of antiapoptotic genes (Bcl-2, DUSP2, and XIAP) and upregulating the levels of proapoptotic genes (cleaved caspase-3 and Bax), ultimately inhibiting the apoptosis of EC cells. There are accumulating evidence supports that EMT plays an important role in cancer cell migration and invasion, which can be regulated by E-Cadherin, N-Cadherin and Vimentin. For example, Angst et al. [33] proposed that the conversion of E-cadherin to N-cadherin is an important reason for activating the EMT process. In addition, upregulation of vimentin activates the TGF-*β* pathway and induces the EMT process [34]. Similarly, in this study, we observed that overexpressed circPOLR1C also reversed the regulation of miR-361-3p on E-cadherin, N-cadherin, and vimentin and promoted the migration and invasion of EC cells.

## 5. Conclusion

In summary, this study confirmed that circPOLR1C, which is highly expressed in EC tissues and cells, promotes xenograft growth, lung metastasis, proliferation, and metastasis yet inhibits the apoptosis of EC cells. The specific mechanism is to regulate the genes related to apoptosis and EMT through adsorption and inhibition of miR-361-3p. To some extent, this study further enriches the basic research arguments of circRNA intervention in EC.

## Figures and Tables

**Table 1:** Primers for qRT-PCR.

Genes	Forward primer (5′-3′)	Reverse primer (5′-3′)
circPOLR1C	CCAAACAACACGGAGACGGA	CCAAACAACACGGAGACGGA
POLR1C	AACGGCATGTCTCGATTGGA	CAGAATCTGCACCCAGCAATG
miR-604	GGACGTCGTATCCAGTGCAA	GTCGTATCCAGTGCGTGTCG
miR-1253	AGAAGATCAGCCTGCAGTCG	GTGTCGTGGAGTCGGCAATT
miR-182	TGGTTCTAGACTTGCCAACTAGT	TGTCGTGGAGTCGGCAATTG
miR-142-5p	CTGTCGTATCCAGTGCAATTGC	GTCGTATCCAGTGCGTGTCG
miR-361-3p	TCCCCCAGGTGTGATTCTGA	TGTCGTGGAGTCGGCAATTG
miR-183	CGAAGGGCCATAAGTCGTATCC	CAGTGCGTGTCGTGGAGTC
Bcl-2	GAACTGGGGGAGGATTGTGG	ACTTCACTTGTGGCCCAGAT
Bax	CAGAGGCGGGGGATGATTG	GAGCTAGGGTCAGAGGGTCA
E-cadherin	CCCGGGACAACGTTTATTAC	GCTGGCTCAAGTCAAAGTCC
N-cadherin	GCTCTCCCTCCCTGTTCC	GGACTCGCACCAGGAGTAATAA
Vimentin	TCCGCACATTCGAGCAAAGA	ATTCAAGTCTCAGCGGGCTC
SH2B1	CTGTGGAGGTGATCAGAGTGG	AGATATCTCGCAAGACCCGC
SF3B3	TGGAGAGAGAGCTGGATGCT	GAACTGCTGGTGGTAGTCCC
DUSP2	TACTTCCTGCGAGGAGGCTT	TAGACAGGAGCCCTGGAGTC
CYR61	ACCAGGACTGTGAAGATGCG	AGCCTGTAGAAGGGAAACGC
XIAP	GTGCGCGCGCTAGAAAAG	TTCTGACCAGGCACGATCAC
FOXO3	TAACTTTGATTCCCTCATCT	TAGGTCTTGTGTCAGTTTGA
U6	CTCGCTTCGGCAGCACATATACT	ACGCTTCACGAATTTGCGTGTC
GAPDH	CCCCGGTTTCTATAAATTGAGC	CTTTCCCCATGGTGTCTGAG

**Table 2:** Correlation between circPOLR1C expression and clinical parameters of esophageal cancer patients.

Characteristics		*n*	circPOLR1C expression		*p* value
			Negative	Positive	
Gender	Male	26	14	12	0.552
	Female	20	9	11	

Age (years)	<60	18	8	10	0.546
	≥60	28	15	13	

Tumor size (cm)	<4	22	14	8	0.077
	≥4	24	9	15	

Differentiation	Well	14	11	3	<0.001
	Moderate	20	12	8	
	Poor	12	0	12	

Tumor invasion (T)	T1-T2	21	15	6	0.008
	T3-T4	25	8	17	

Lymph node metastasis	Absent	16	7	9	0.536
	Present	30	16	14	

TNM stage	I + II	18	11	7	0.227
	III + IV	28	12	16	

## Data Availability

The analyzed datasets generated during the study are available from the corresponding author on reasonable request.

## Supplementary Materials

Supplementary Materials Supplementary figures: Supplementary Figure 1. The regulation of the miR-361-3p mimic and overexpressed circPOLR1C on apoptosis-related genes, epithelial-mesenchymal transition (EMT)-related genes, and oncogenes. (a, b) The effects of the miR-361-3p mimic and circPOLR1C on the mRNA levels of apoptosis- and EMT-related genes were detected by qRT-PCR. GAPDH was employed as the internal reference. (c)–(f) The effects of the miR-361-3p mimic and circPOLR1C on the protein levels of apoptosis- and EMT-related genes were assessed by Western blot. GAPDH was applied as the internal reference. (g)–(h) QRT-PCR was used to screen different oncogenes and apoptotic signaling pathways that miR-361-3p and circPOLR1C might be involved in. GAPDH was utilized as the internal reference. (i, j) Western blot was used to detect the protein expression of apoptotic signaling pathway. GAPDH served as the internal reference. All experiments were independently performed in triplicate. ^*∗∗*^*p* < 0.01; ^*∗∗∗*^*p* < 0.001 vs. NC + MC; ^ ^ ^*p* < 0.001 vs. NC + M; ^##^*p* < 0.01; ^###^*p* < 0.001 vs. circPOLR1C + MC. Supplementary Figure 2. Effects of siDUSP2, siXIAP, and siBCL2 on the invasion and apoptosis of EC cells. (a, b) The effects of siDUSP2, siXIAP, and siBCL2 on the invasion of EC cells were evaluated by the transwell assay under 250x magnification. (c, d) The effects of siDUSP2, siXIAP, and siBCL2 on the apoptosis of EC cells were determined by flow cytometry. All experiments were independently performed in triplicate. ^*∗*^*p* < 0.05; ^*∗∗*^*p* < 0.01; ^*∗∗∗*^*p* < 0.001 vs. siNC.

## References

[1] Ferlay J., Soerjomataram I., Dikshit R. (2015). Cancer incidence and mortality worldwide: sources, methods and major patterns in GLOBOCAN 2012. *International Journal of Cancer*.

[2] Chen W., Zheng R., Baade P. D. (2016). Cancer statistics in China, 2015. *CA: A Cancer Journal for Clinicians*.

[3] Chen W. Q., Zeng H. M., Zheng R. S., Zhang S. W., He J., He J. (2012). Cancer incidence and mortality in China, 2007. *Chinese Journal of Cancer Research*.

[4] Wu X., Chen V. W., Ruiz B., Andrews P., Su L. J., Correa P. (2006). Incidence of esophageal and gastric carcinomas among American Asians/Pacific Islanders, whites, and blacks: subsite and histology differences. *Cancer*.

[5] Jemal A., Center M. M., DeSantis C., Ward E. M. (2010). Global patterns of cancer incidence and mortality rates and trends. *Cancer Epidemiology, Biomarkers & Prevention*.

[6] Burki T. K. (2017). Definitions of oesophageal cancer. *The Lancet Oncology*.

[7] Hou X., Wen J., Ren Z., Zhang G. (2017). Non-coding RNAs: new biomarkers and therapeutic targets for esophageal cancer. *Oncotarget*.

[8] Patop I. L., Wüst S., Kadener S. (2019). Past, present, and future of circRNAs. *EMBO Journal*.

[9] Sang M., Meng L., Sang Y. (2018). Circular RNA ciRS-7 accelerates ESCC progression through acting as a miR-876-5p sponge to enhance MAGE-A family expression. *Cancer Letters*.

[10] Hu X., Wu D., He X. (2019). CircGSK3*β* promotes metastasis in esophageal squamous cell carcinoma by augmenting *β*-catenin signaling. *Molecular Cancer*.

[11] Thiffault I., Wolf N. I., Forget D. (2015). Recessive mutations in POLR1C cause a leukodystrophy by impairing biogenesis of RNA polymerase III. *Nature Communications*.

[12] Kraoua I., Karkar A., Drissi C. (2019). Novel POLR1C mutation in RNA polymerase III-related leukodystrophy with severe myoclonus and dystonia. *Mol Genet Genomic Med*.

[13] Ghesh L., Vincent M., Delemazure A. S. (2019). Autosomal recessive Treacher Collins syndrome due to POLR1C mutations: report of a new family and review of the literature. *American Journal of Medical Genetics, Part A*.

[14] Pinto J. A., Araujo J., Cardenas N. K. (2016). A prognostic signature based on three-genes expression in triple-negative breast tumours with residual disease. *Npj Genomic Medicine*.

[15] Fan L., Cao Q., Liu J., Zhang J., Li B. (2019). Circular RNA profiling and its potential for esophageal squamous cell cancer diagnosis and prognosis. *Molecular Cancer*.

[16] Domingues S., Moreira R. N., Andrade J. M. (2015). The role of RNase R in trans-translation and ribosomal quality control. *Biochimie*.

[17] Schmittgen T. D., Livak K. J. (2008). Analyzing real-time PCR data by the comparative C(T) method. *Nature Protocols*.

[18] Yu J., Xu Q. G., Wang Z. G. (2018). Circular RNA cSMARCA5 inhibits growth and metastasis in hepatocellular carcinoma. *Journal of Hepatology*.

[19] Kim B. (2017). Western blot techniques. *Methods in Molecular Biology*.

[20] Sihvo E., Anttonen A., Huuhtanen R. (2014). [Treatment of esophageal cancer]. *Duodecim*.

[21] Lan X., Liu X., Sun J., Yuan Q., Li J. (2019). CircRAD23B facilitates proliferation and invasion of esophageal cancer cells by sponging miR-5095. *Biochemical and Biophysical Research Communications*.

[22] Li R. C., Ke S., Meng F. K. (2018). CiRS-7 promotes growth and metastasis of esophageal squamous cell carcinoma via regulation of miR-7/HOXB13. *Cell Death & Disease*.

[23] Liu J., Xue N., Guo Y. (2019). CircRNA_100367 regulated the radiation sensitivity of esophageal squamous cell carcinomas through miR-217/Wnt3 pathway. *Aging (Albany NY)*.

[24] Chen W., Wang J., Liu S. (2016a). MicroRNA-361-3p suppresses tumor cell proliferation and metastasis by directly targeting SH2B1 in NSCLC. *Journal of Experimental & Clinical Cancer Research*.

[25] Hu J., Li L., Chen H. (2018). MiR-361-3p regulates ERK1/2-induced EMT via DUSP2 mRNA degradation in pancreatic ductal adenocarcinoma. *Cell Death & Disease*.

[26] Hua B., Li Y., Yang X., Niu X., Zhao Y., Zhu X. (2020). MicroRNA-361-3p promotes human breast cancer cell viability by inhibiting the E2F1/P73 signalling pathway. *Biomedicine & Pharmacotherapy*.

[27] Chen L., Nan A., Zhang N. (2019). Circular RNA 100146 functions as an oncogene through direct binding to miR-361-3p and miR-615-5p in non-small cell lung cancer. *Molecular Cancer*.

[28] Wang J., Li H., Liang Z. (2019). <p>circ-MYBL2 serves as A sponge for miR-361-3p promoting cervical cancer cells proliferation and invasion<</p>. *OncoTargets and Therapy*.

[29] Xia F., Chen Y., Jiang B., Bai N., Li X. (2020). Hsa_circ_0011385 accelerates the progression of thyroid cancer by targeting miR-361-3p. *Cancer Cell International*.

[30] Cheung C. H. A., Chang Y. C., Lin T. Y., Cheng S. M., Leung E. (2020). Anti-apoptotic proteins in the autophagic world: an update on functions of XIAP, Survivin, and BRUCE. *Journal of Biomedical Science*.

[31] Hsiao K. Y., Chang N., Tsai J. L., Lin S. C., Tsai S. J., Wu M. H. (2017). Hypoxia-inhibited DUSP2 expression promotes IL-6/STAT3 signaling in endometriosis. *American Journal of Reproductive Immunology*.

[32] Hassan M., Watari H., AbuAlmaaty A., Ohba Y., Sakuragi N. (2014). Apoptosis and molecular targeting therapy in cancer. *BioMed Research International*.

[33] Angst B. D., Marcozzi C., Magee A. I. (2001). The cadherin superfamily: diversity in form and function. *Journal of Cell Science*.

[34] Yu S., Yan C., Yang X. (2016). Pharmacoproteomic analysis reveals that metapristone (RU486 metabolite) intervenes E-cadherin and vimentin to realize cancer metastasis chemoprevention. *Scientific Reports*.

